# Segregation of Unknown Odors From Mixtures Based on Stimulus Onset Asynchrony in Honey Bees

**DOI:** 10.3389/fnbeh.2019.00155

**Published:** 2019-07-12

**Authors:** Aarti Sehdev, Paul Szyszka

**Affiliations:** ^1^Department of Biology, Neurobiology, University of Konstanz, Konstanz, Germany; ^2^Department of Zoology, University of Otago, Dunedin, New Zealand

**Keywords:** olfaction, insects, odor mixtures, odor-background segregation, blind source separation, temporal stimulus cues

## Abstract

Animals use olfaction to search for distant objects. Unlike vision, where objects are spaced out, olfactory information mixes when it reaches olfactory organs. Therefore, efficient olfactory search requires segregating odors that are mixed with background odors. Animals can segregate known odors by detecting short differences in the arrival of mixed odorants (stimulus onset asynchrony). However, it is unclear whether animals can also use stimulus onset asynchrony to segregate odorants that they had no previous experience with and which have no innate or learned relevance (unknown odorants). Using behavioral experiments in honey bees, we here show that stimulus onset asynchrony also improves segregation of those unknown odorants. The stimulus onset asynchrony necessary to segregate unknown odorants is in the range of seconds, which is two orders of magnitude larger than the previously reported stimulus asynchrony sufficient for segregating known odorants. We propose that for unknown odorants, segregating odorant A from a mixture with B requires sensory adaptation to B.

## Introduction

Natural olfactory stimuli are typically mixtures of many different odorants from different sources, which mix together in turbulent plumes (Murlis et al., 1992; Kree et al., 2013; Celani et al., 2014; Riffell et al., 2014; Soltys and Crimaldi, 2015; Erskine et al., 2019). Previous studies suggested that animals perceive odorant mixtures synthetically, that is, they perceive a mixture as a perceptual unit rather than as a list of individual odorants (humans: Jinks and Laing, 1999; squirrel monkeys: Laska and Hudson, 1993; rats: Staubli et al., 1987; spiny lobsters: Lynn et al., 1994, honey bees: Chandra and Smith, 1998; Smith, 1998; Deisig et al., 2001). But, animals often need to perceptually segregate mixed odorants that come from different sources (analytic mixture perception; Hopfield, 1991; Stevenson and Wilson, 2007).

Due to the turbulent structure of plumes, odorants that originate from the same source fluctuate with stable relative concentration proportions, forming a homogeneous stream of plumes, whereas odorants from different sources will have varying relative concentration proportions, creating a heterogeneous stream of plumes (Hopfield, 1991; Kree et al., 2013; Celani et al., 2014; Riffell et al., 2014; Soltys and Crimaldi, 2015; Erskine et al., 2019). Therefore, homogeneous and heterogeneous plumes could provide the animal with information about the number of odor sources and which odorants belong to the same odor source. In particular, odorants that arise from a single source would arrive at the olfactory organ synchronously, whereas odorants that arise from multiple sources would differ in their arrival times (Hopfield, 1991). Accordingly, invertebrates can use both spatial and temporal information from odor plumes for odor-background segregation (spatial: Hopfield and Gelperin, 1989; Baker et al., 1998; Andersson et al., 2011; Weissburg et al., 2012; temporal: Szyszka et al., 2012; Saha et al., 2013; Sehdev et al., 2019). Remarkably, tobacco hawk moths can segregate odorant sources separated by only 1 mm (Baker et al., 1998), and honey bees can use odorant onset asynchronies as short as 6 ms to segregate a known odorant (odorant with innate or learned valence) from another odorant (Szyszka et al., 2012).

All previous studies that have investigated odor segregation based on temporal stimulus cues have in common that the odorants either had an innate valence (Baker et al., 1998; Nikonov and Leal, 2002; Andersson et al., 2011; Weissburg et al., 2012) or a learned valence (Hopfield and Gelperin, 1989; Szyszka et al., 2012; Saha et al., 2013; Sehdev et al., 2019), and it is currently unknown whether animals can use temporal stimulus cues to segregate odorants that have no innate or learned valence and that the animals had no previous experience with. We herein refer to those odorants as unknown odorants.

To test whether stimulus onset asynchrony aids segregating an unknown odorant from a mixture, we trained honey bees in a classical conditioning assay (Bitterman et al., 1983) to associate a mixture of a single component odor A and a four-component odor B with a sucrose reward. B started either before A (asynchronous mixture) or simultaneously with A (synchronous mixture). Thus, during conditioning, bees could never encounter A alone. We then tested whether bees had segregated A from B during conditioning by testing bees’ response to A. Odorant onset asynchronies of 20 or 5 s aided odor segregation, but odorant onset asynchronies of 1 or 0.2 s did not. These data suggest that bees can use stimulus onset asynchrony to segregate an unknown odorant from mixtures, but not on the millisecond timescale seen for known odorants (Szyszka et al., 2012).

## Materials and Methods

### Animals

Worker honey bees (*Apis mellifera*) were collected from the entrance of outdoor hives at University of Konstanz between 09:00 and 12:00 between June 2016 and December 2016. Bees were anesthetized using ice and fixed into a holder using sticky tape, so that proboscis, antennae and mandibles were freely movable. The fixed bees were left undisturbed and starved for 3 h before the conditioning procedure started, in order to encourage a response to a sucrose reward. We only used bees that showed responses to sucrose when presented at the antennae. Up to 16 bees were conditioned in parallel in each experimental session.

### Odorant Delivery

For odorant A, we used either 1-hexanol or nonanal. We chose these odorants based on their perceived dissimilarity to each other (25%–30% of bees generalize between 1-hexanol and nonanal, Guerrieri et al., 2005). We mixed the single-component odor A with a four-component odor blend B, comprised of 1-octanol, heptanal, hexanal and 2-hexanone. We chose these odorants for B to make the odor segregation task challenging, as they activate a set of olfactory receptor neurons that largely overlaps with the set of receptor neurons activated by the A odorants. For example, in case of the A odorant 1-hexanol, glomerulus 28 and 38 are the strongest responding glomeruli, and these glomeruli also respond strongly to three (glomerulus 28) or one (glomerulus 38) of the four B odorants (Sachse et al., 1999). In the case of A odorant nonanal, glomerulus 33 and 17 are the strongest responding glomeruli and these glomeruli also respond strongly to four (glomerulus 33) or two (glomerulus 17) of the four B odorants. All odorants were from Sigma-Aldrich.

Odorants were delivered to the bees’ antennae by a custom-made olfactory stimulator, as described in Raiser et al. (2016). We used pure odorants, which were kept in 20 ml glass vials (Schmidlin) sealed with a Teflon septum. The headspace of odorized air was extracted and drawn into an air dilution system using flowmeters (112-02GL, Analyt-MTC) and an electronic pressure control (35898; Analyt-MTC). The olfactory stimulator used three channels: one for nonanal, one for 1-hexanol and one for the four-component blend B. To apply B, the four odorants were mixed by connecting the four odorant vials in series and passing 50 ml/min of air through their headspaces, allowing the four odorants to mix. For each odor channel, the rate of air flow was 300 ml/min, with 50 ml/min of odorant combined with a dilution of 250 ml/min of clean air. The total air flow at the outlet of the stimulator was 4.7 L/min, with an airspeed of 1 m/s. The outlet of the stimulator had an inner diameter of 1 cm and was positioned 1 cm in front of the center of the bee’s head. Note that the concentration of B was likely higher than the concentration of A since we used four odorants as B and only one as A, and the vapor pressures of the B odorants were up to one order of magnitude higher than those of the A odorants (A odorants: 1-hexanol: 0.1 kPa, nonanal: 0.05 kPa; B odorants: 1-octanol: 0.001 kPa, heptanal: 0.5 kPa, hexanal: 1.5 kPa, 2-hexanone: 1.5 kPa; all at 25°C).

The valves of the olfactory stimulator were controlled by a compact RIO system equipped with a digital I/O module NI-9403 (National Instruments, Austin, TX, USA) using software written by Stefanie Neupert in LabVIEW 2011 SP1 (National Instruments, Austin, TX, USA). The odorant vials were constantly flushed with air throughout the experiment so that the headspace concentration reached a dynamic steady state. To generate the synchronous mixture, the two odor channels delivering A or B were opened simultaneously. To generate the asynchronous mixtures, the two odor channels were opened with a time delay. All odorants were removed from the bees* via* an exhaust placed behind the bee.

### Conditioning Paradigm

All experiments are based on classical absolute conditioning by pairing an olfactory stimulus (conditioned stimulus) with a sucrose reward (unconditioned stimulus; Bitterman et al., 1983). The sucrose reward was 1.25 M sucrose-water solution and was applied by a metal pinhead (1 mm diameter) that was dipped into the sucrose. The sucrose reward was presented for 4 s, first to the antennae to induce the proboscis extension response and then to the proboscis to allow for feeding. Bees were conditioned for five trials and the inter-stimulus interval was approximately 14 min. The time between the final conditioning trial and the test was 30 min. Overall, 486 bees were tested.

During conditioning, fixed bees were placed in front of the stimulator where they were left to acclimatize to the airflow for 20 s. After this, B started, and after a delay of either 20 s (B20A), 5 s (B5A), 1 s (B1A) or 0.2 s (B0.2A), A started. For the synchronous mixture AB, B and A started simultaneously. Three seconds after the onset of A, the sucrose reward was presented. LEDs were used to indicate to the experimenter when to present sucrose to the bees and when the different odorants were released from the stimulator valves.

All experiments were balanced, such that odorant A and the novel odorant N were equally often 1-hexanol or nonanal, and the data were pooled. This procedure minimizes non-associative effects of the conditioning, such as odorant-specific changes in hedonic value, generalization or sensitization (Quinn et al., 1974). During the test, each bee received a single stimulation with A and a single stimulation with B. The inter-stimulus interval was 14 min, and the order of A and N stimulations was balanced across bees. B was not presented during the test.

### Quantifying Conditioned Response

We monitored the conditioned response as the occurrence of the proboscis extension reflex during the odorant stimulation. We counted a proboscis extension reflex only when the proboscis was not extended before odorant onset and when it was extended horizontally during the odorant stimulation not overlapping with the sucrose reward. The conditioned response was documented in a binary form.

For each conditioning trial, the percentage of bees that showed proboscis extension reflex to each odorant was recorded. For those experiments where the response to B could be discerned visually from the response to A (B20A, B5A), the proboscis extension reflex was recorded for both A and B. During the test, the presence or absence of proboscis extension reflex to the conditioned odorant was recorded as a 1 or 0 respectively. To assess the associative memory performance, we separated responses into “correct” and “incorrect” responses. Bees that responded to the A during the test but not the novel odorant N were given a score of 1; all other responses were deemed incorrect and given a score of 0. The proportion of correct responses was then statistically compared between groups.

Bees that died during the experiment, or those that did not respond to sucrose when delivered to the antennae at the end of the experiment, were discarded from the analysis.

### Statistical Analysis

For all data analysis, R version 3.5.2 was used (R Core Team, 2017). All statistical tests were performed using Bayesian data analysis, based on Korner-Nievergelt et al. (2015).

To investigate the effect of odorant A and novel odorant N on conditioned response, we used a binomial generalized linear model, with conditioned response as the binary response variable (1 = conditioned response, 0 = no conditioned response). We used the logistic regression (logit) link function. Odorant A and novel odorant N were used as explanatory variables. We used an improper prior distribution (flat prior) and simulated 100,000 values from the posterior distribution of the model parameters using the function “sim” from the package “arm.” The means of the simulated values from the posterior distributions of the model parameters were used as estimates and the 2.5% and 97.5% quantiles as the lower and upper limits of the 95% credible intervals. To test for differences between conditioned responses to odorant A and novel odorant N, we compared the probabilities of conditioned response by calculating the proportion of simulated values from the posterior distribution that were larger in odorant A than in the novel odorant N. A posterior probability of, for example, 0.953 for the comparison between odorant A and the novel odorant N [*p(A > N)* = 0.953] means that one can be 95% certain that the probability of conditioned response is greater for A than for N. We declared an effect to be significant if the proportion was greater than 0.95.

To investigate the effect of mixtures and single odorants or of synchronous and asynchronous mixtures on correct responses, we used the same analysis as above, with the appropriate explanatory variables.

## Results

To investigate bees’ capability to segregate an unknown odorant from a mixture, we conditioned fixed bees to associate a mixture of an odorant A (either 1-hexanol or nonanal), and a four-component blend B (1-octanol, heptanal, hexanal and 2-hexanone) with a sucrose reward. Sucrose was always presented 3 s after the onset of A. Thirty minutes after the last conditioning trial, the conditioned response to A alone or to a novel odorant (N; either 1-hexanol or nonanal) was tested in each bee. To eliminate between-session variability, all data shown in a given panel of a figure were collected in parallel during the same experimental sessions. Accordingly, data points should be compared within panels, but not between panels (original data are available in Supplementary Data Sheet 1).

### Five Seconds Onset Asynchrony or Longer Improves Segregation of Unknown Odorants

**Figure 1:**
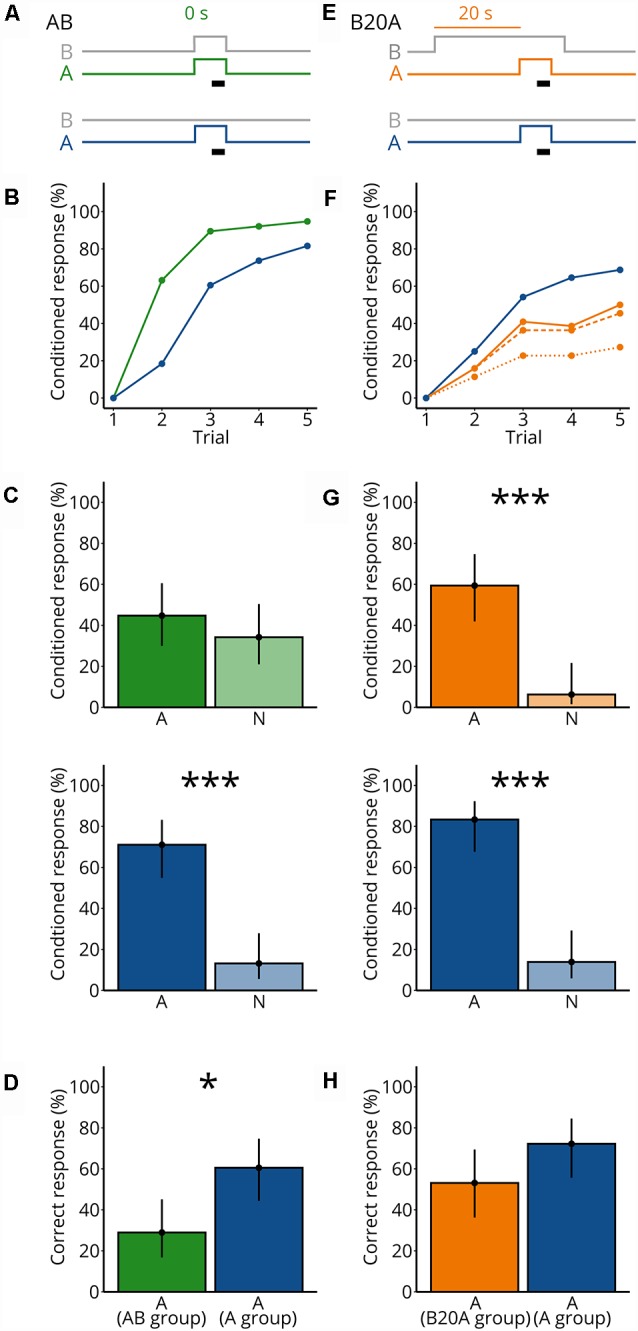
Mixing an unknown odorant A with an odorant blend B impairs detection of A. **(A)** Valve states for creating odorant pulses for the synchronous mixture AB and the control A. For AB, both A and the background B (gray) were turned on synchronously and were presented for 7 s. The black bar indicates the 4 s when the sucrose reward was given. For the control A, A (blue) was presented for 7 s. B (gray) was not presented. **(B)** Each bee received five rewarded conditioned trials either with AB (green) or A (blue); the percentage of bees responding to the odorants is shown. *N* = 38 bees conditioned to AB and 38 bees conditioned to A. **(C)** During the two test trials, each bee was stimulated with A and a novel odorant N. Percentage of bees responding to A and to N for bees conditioned to AB (green) and bees conditioned to A (blue). Points represent means and vertical lines represent 95% credible intervals for all panels in this figure. Stars indicate significant differences between means for all panels in this figure (*probability for a difference between both means *p* > 0.95; ^***^p > 0.999). **(D)** Percentage of correctly responding bees during the test (response to A but not to N) for bees conditioned to AB (green) and A (blue). **(E)** Valve states for creating odorant pulses for the asynchronous mixture B20A and the control A. For B20A, A (orange) was turned on 20 s after the background B (gray). A was presented for 7 s. B ended 3 s after A ended. Same control as in **(A)**. **(F)** Same as in **(B)**, but for B20A. Percentage of bees responding to only A (dotted line), only A or to both A and B within the same trial (dashed line), and to A and/or B (solid line) for bees conditioned to B20A (orange) and A alone (blue). *N* = 32 bees conditioned to B20A and 36 bees conditioned to A. **(G)** Same as in **(C)**, but for B20A. **(H)** Same as in **(D)**, but for B20A.

We firstly reassessed the finding of previous studies, that mixing odorants impairs bees’ recognition of the individual odorants (Chandra and Smith, 1998; Smith, 1998; Deisig et al., 2001). We conditioned bees to associate a synchronous mixture of A and B (AB, where A and B start at the same time) with a sucrose reward (Figure 1A). A second group was conditioned to A without B (Figure 1A).

By the fifth conditioning trial, 95% of bees conditioned to AB responded and 82% of the bees conditioned to A alone responded (Figure 1B). To test whether the bees associated odorant A with the sucrose reward during conditioning, we then recorded the response to A or to a novel odorant N in the absence of sucrose (the order of A and N was balanced across bees; Figure 1C). Significantly more bees responded to A than to N in the group conditioned to A [*p(A > N)* > 0.999], but not in the group conditioned to AB [*p(A > N)* = 0.826], indicating that bees recognize A as an odor predictive for the sucrose reward when conditioned to A but not when conditioned to AB. During the test, a bee could respond in several ways. A bee could respond correctly, that is showing a conditioned response to A and not to N; alternatively, a bee could generalize by responding to both odorants, or show a lack of response to A. To determine whether the groups conditioned to A or to the synchronous mixture differed in their expression of correct responses, we calculated the percentage of bees’ correct responses in the test (Figure 1D). The percentage of correctly responding bees was lower when conditioned to the synchronous mixture AB than to A alone {*p[A(A group) > A(AB group)*] = 0.997}, confirming earlier studies showing that mixing odorants impairs the recognition of individual odorant components, indicating that the perception of odorant mixture is partly synthetic (Chandra and Smith, 1998; Smith, 1998; Deisig et al., 2001).

Because stimulus onset asynchrony can improve odor segregation for known odorants (Hopfield and Gelperin, 1989; Baker et al., 1998; Andersson et al., 2011; Szyszka et al., 2012; Sehdev et al., 2019), we asked whether presenting A and B as an asynchronous mixture would still impair the recognition of A. We presented B for 30 s and after 20 s we added A for 7 s (B20A; Figure 1E). B always stopped 3 s after A stopped, to make sure that bees would never encounter A without B. The sucrose reward was presented 4 s after the onset of A (Figure 1E). As a control, bees were conditioned to A without the mixture. By the fifth conditioning trial, 27% of the bees conditioned to B20A responded to A only and 45% responded to both A and B, and 50% responded to A and/or B, and 69% of the bees conditioned to A alone responded to A (Figure 1F). During the test, more bees conditioned to B20A responded to A than to N [*p(A > N)* > 0.999], as did bees conditioned to A [*p(A > N)* > 0.999; Figure 1G], showing that bees could segregate A from B20A during the conditioning. Moreover, the percentage of correctly responding bees did not significantly differ when conditioned to B20A than to A alone {*p[A(A group) > A(B20A group)]* = 0.947} (Figure 1H). These results suggest that bees could segregate an odorant A in the asynchronous mixture B20A but not in the synchronous mixture AB.

**Figure 2:**
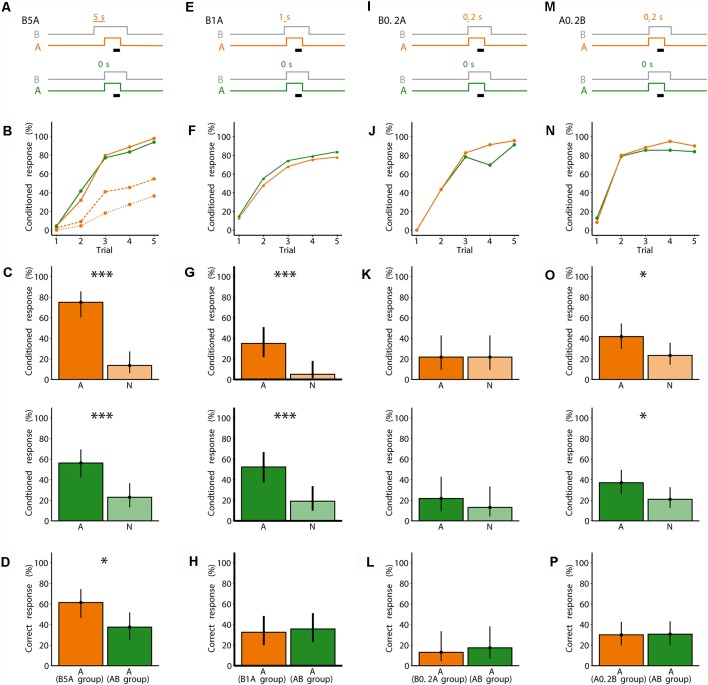
Five seconds onset asynchrony between an odorant blend B and a following unknown odorant A improves odor segregation. **(A)** Valve states for creating odorant pulses for the asynchronous mixture B5A and the synchronous mixture AB. For B5A, A turned on 5 s after B (gray). A was presented for 7 s. B ended 3 s after A ended. The black bar indicates the 4 s when the sucrose reward was given. For AB, both A and the background B (gray) were turned on synchronously. **(B)** Each bee received five rewarded training trials either with B5A (orange) or AB (green). Percentage of bees responding to only A (dotted line), only A or to both A and B within the same trial (dashed line), and to A and/or B (solid line). *N* = 44 bees conditioned to B5A and 48 bees conditioned to AB. **(C)** During the two test trials, bees were stimulated with A or a novel odorant N. Percentage of response to A and to N for bees conditioned to B5A (orange) and bees conditioned to AB (green). Points represent means and vertical lines represent 95% credible intervals for all panels in this figure. Stars indicate significant differences between means for all panels in this figure (*probability for a difference between both means *p* > 0.95; ^***^p > 0.999). **(D)** Percentage of correctly responding bees during the test (response to A but not to N) for bees conditioned to B5A (orange) and A (green). **(E)** Same as in **(A)**, but for B1A. A turned on 1 s after B. **(F)** Same as in **(B)**, but for B1A as asynchronous mixture. Separate responses to A and B were not distinguishable. *N* = 40 bees conditioned to B1A and 42 bees conditioned to AB. **(G)** Same as in **(C)**, but for B1A. **(H)** Same as in **(D)**, but for B1A. **(I)** Same as in **(A)**, but for B0.2A. A turned on 0.2 s after B. **(J)** Same as in **(F)**, but for B0.2A as asynchronous mixture. *N* = 23 bees conditioned to B0.2A and 23 bees conditioned to AB. **(K)** Same as in **(C)**, but for B0.2A. **(L)** Same as in **(D)**, but for B0.2A. **(M)** Same as in **(A)**, but for A0.2B. A turned on 0.2 s before B. **(N)** Same as in **(F)**, but for A0.2B as asynchronous mixture. *N* = 60 bees conditioned to A0.2B and 62 bees conditioned to AB. **(O)** Same as in **(C)**, but for A0.2B. **(P)** Same as in **(D)**, but for A0.2B.

To determine the limit of stimulus onset asynchrony that bees could use for odor segregation, we investigated three onset asynchronies between B and a following odorant A: 5 s (B5A), 1 s (B1A) and 0.2 s (B0.2A; Figure 2A). We compared bees’ capability to segregate odorant A against a parallel group of bees that were conditioned to the synchronous mixture AB (A and B had a synchronous onset, but different to the experiment in Figure 1A, B stopped 3 s after A stopped). During the fifth conditioning trial, 36% of bees conditioned to B5A responded to A only, 55% responded to A and B, and 98% responded to A and/or B (Figure 2B). In comparison, 93% of bees conditioned to AB responded to AB (Figure 2B).

The bees conditioned to B5A responded more to A than to N during the test [*p(A > N)* > 0.999], showing that they were able to segregate A from B during conditioning (Figure 2C). The AB-conditioned bees also responded more to A than to N [*p(A > N)* = 0.999; Figure 2C], showing that they also could segregate A from B. However, bees conditioned to B5A showed more correct responses than bees conditioned to AB {*p[A(B5A group) > A(AB group)]* = 0.989} (Figure 2D). This shows that stimulus onset asynchrony of 5 s improves segregating a odorant A from an asynchronous mixture (B5A) as compared to synchronous presentation of both odorant A and the mixture (AB).

### One Second Onset Asynchrony or Shorter Does Not Improve Segregation of Unknown Odorants

Bees conditioned to the asynchronous mixtures B1A and B0.2A (Figures 2E,I) showed similar responses to the synchronous mixtures AB during conditioning. The onset asynchrony between B and A of 1 s or less was too short to determine whether their conditioned response was to A or to B. During the fifth conditioning trial, 78% and 83% responded to B1A and AB, respectively (Figure 2F), and 96% and 91% responded to B0.2A and AB, respectively (Figure 2J).

During the test, bees of the B1A group and the parallel AB control group showed a higher percentage of responses to A than to N [group B1A: *p(A > N)* = 0.998; group AB: *p(A > N)* > 0.999; Figure 2G]. However, bees of the B0.2A group and the parallel AB control group showed no difference in their responses to A and N [group B0.2A *p(A > N)* = 0.501; group AB: *p(A > N)* = 0.781; Figure 2K], indicating that neither bees of the B0.2A group nor of the parallel AB control group segregated A during conditioning. Note that bees’ capability to segregate A from AB during conditioning differed between the experimental sessions shown in Figures 2G,K. This indicates that bees’ capability to segregate an odorant from a mixture may vary and depend on factors other than the experimentally controlled factors (e.g., season, bees’ age). For both the B1A group and the B0.2A group, there was no difference in the proportion of correct responses to the AB-conditioned control group {*p[A(B1A group) > A(AB group)]* = 0.381; Figure 2H; *p[A(B0.2A group) > A(AB group)]* = 0.343; Figure 2L}.

In summary, in the experiments in Figure 2, bees were able to segregate A from B when conditioned to an asynchronous or synchronous mixture (except for B0.2A). However, bees showed a higher percentage of correct responses for an onset asynchrony of 5 s between B and A, and not for shorter onset asynchronies. Although the AB-conditioned bees were also able to segregate A, the proportion of correct responses in the test was either lower than the proportion of correct responses from bees conditioned to the asynchronous mixture (B5A), or there was no difference between the proportion of correct responses in the test between bees conditioned to AB or to the asynchronous mixtures (B1A, B0.2A). The finding that bees could segregate A from AB in Figure 2, but not in Figure 1, could be due to the fact, that in Figure 2 the offset of A was 3 s earlier than that of B, while in Figure 1 the offsets of A and B were synchronous. Note however, that we did not investigate further the effect offset asynchrony on odor segregation. In all of the previous experiments, during conditioning, B was presented before A, thus bees could never experience A alone. We therefore asked whether presenting A before B would improve segregation of A from B. We conditioned bees using an asynchronous mixture of A and B, in which B started 0.2 s after the onset of A (A0.2B; Figure 2M). Thus, bees experienced odorant A alone for 0.2 s before the onset of B. Again, we used the synchronous mixture AB as a control. During the fifth conditioning trial, 90% of the bees responded to A0.2B and 83% responded to AB (Figure 2N). In the test, bees conditioned to A0.2B showed a higher percentage of conditioned responses to A than to N [*p(A > N)* = 0.983], as did the bees conditioned to AB [*p(A > N)* = 0.975; Figure 2O]. However, the percentage of correct responses during the test was not different between the two groups {*p[A(A0.2B group) > A(AB group)]* = 0.471} (Figure 2M). Therefore, even encountering odorant A alone for a short time does not improve its segregation from a mixture.

## Discussion

We asked whether honey bees can use stimulus onset asynchrony to segregate an unknown odorant A (an odorant that has no innate or learned valence and that the animals had no previous experience with) from a mixture. We found that stimulus onset asynchronies of 5 s or more improved odor segregation, while stimulus onset asynchronies of 1 s or less did not. This timescale for segregating unknown odorants based on stimulus onset asynchrony is at least two orders of magnitude slower than for segregating known odorants (Baker et al., 1998; Szyszka et al., 2012; Sehdev et al., 2019). These results suggest that the neural mechanisms for odor segregation based on stimulus onset asynchrony differ for known and unknown odorants.

### Perception of Odorant Mixtures

The finding that bees were impaired in recognizing the odorant A in a mixture with B (Figures 1A–D) is in line with previous studies which suggested that honey bees perceive odorant mixtures partly synthetically (Chandra and Smith, 1998; Smith, 1998; Müller et al., 2000; Deisig et al., 2001, 2003). However, there is also evidence for analytic mixture perception in bees: when bees are conditioned to a mixture and afterward are tested with the single odorants, they respond to most of the single odorants (Laloi et al., 1999; Reinhard et al., 2010). Analytic mixture perception has also been demonstrated in blocking experiments, in which previous conditioning to odorant A reduces conditioning to B during conditioning with AB because A already predicts the reward (Smith and Cobey, 1994; but see Gerber and Ullrich, 1999 for an opposing view).

The impairment of bees’ capability to segregate an odorant A from a mixture with B could reflect an impaired detection of odorant A due to synthetic mixture perception, or it could reflect an impaired learning of odorant A due to overshadowing (Pavlov, 1927). In honey bees, the potential of one odorant to overshadow another odorant increases with its concentration (Pelz et al., 1997; Reinhard et al., 2010). In our setup, the concentration of B likely was higher than the concentration of A because we used a blend of 4 odorants as B and only one odorant as A (see “Materials and Methods” section), and the vapor pressures of the B odorants were up to one order of magnitude higher than those of the A odorants. It is therefore plausible to assume that B overshadowed A and was learned better than A.

### Odor Segregation Based on Relational Stimuli

Segregating an unknown odorant from a mixture is a blind source separation problem, and solving it requires more information than just the chemical odorant identity (Hendin et al., 1994). The physics of odorant dispersion adds relational information to the chemical odorant identity, as odorants from the same source form plumes with relatively stable odorant concentration proportions (homogeneous plumes), while odorants from different sources form plumes with variable odorant concentration proportions (heterogeneous plumes; Hopfield, 1991; Kree et al., 2013; Celani et al., 2014; Riffell et al., 2014; Soltys and Crimaldi, 2015; Erskine et al., 2019). Indeed, animals can use these relational stimuli to detect whether odorants originate from the same or different sources. For example, honey bees can segregate an unknown odorant A from a mixture ABC when the concentrations of B and C vary from trial to trial (Wright and Smith, 2004). Similarly, mice can segregate an unknown odorant from a mixture whose composition varies from trial to trial (Rokni et al., 2014).

Besides this trial-to-trial variability of odorant concentration or composition, animals can also use stimulus onset asynchrony between mixed odorants for segregating odorants from mixtures (Hopfield and Gelperin, 1989; Baker et al., 1998; Andersson et al., 2011; Szyszka et al., 2012; Weissburg et al., 2012; Saha et al., 2013; Sehdev et al., 2019). However, so far, this capability has only been demonstrated for known odorants that had either an innate or learned valence.

Our data suggest that honey bees can use stimulus onset asynchrony also for segregating unknown odorants. However, the required stimulus onset asynchrony for segregating an unknown odorant is in the range of seconds rather than in the range of milliseconds as is the case for known odorants (Baker et al., 1998; Szyszka et al., 2012; Sehdev et al., 2019).

### Neural Mechanisms for Segregating Known vs. Unknown Odorants

We assessed bees’ capability to segregate an unknown odorant A from a mixture by measuring bees’ capability to associate A with a sucrose reward, while A was presented in a mixture with odor B. In this paradigm, bees required a longer stimulus onset asynchrony for segregating the unknown odorant A as compared to segregating a known odorant from a mixture, seen in previous studies (Baker et al., 1998; Andersson et al., 2011; Szyszka et al., 2012; Saha et al., 2013; Sehdev et al., 2019). This suggests that the olfactory temporal resolution is lower during associative learning of an unknown odorant than during the recognition of a known odorant.

The olfactory temporal resolution during associative odor-reward learning could be limited by the temporal resolution of: (a) the neural encoding of the odor; (b) the neural encoding of the reward; or (c) the molecular processes of odor-reward memory formation. The temporal resolution of neural odor encoding is limited by the time window over which the olfactory system needs to integrate odor-evoked neural responses, and this integration time window depends on the range of response latencies across olfactory neurons (Jeanne and Wilson, 2015; Egea-Weiss et al., 2018). In honey bees, odor-evoked response latencies in projection neurons of the antennal lobe generally differ by less than 1 s (Müller et al., 2002; Krofczik et al., 2009; Brill et al., 2013; Paoli et al., 2018). This suggests that the honey bee olfactory system should require less than 1 s to integrate odor-evoked neural responses for identifying an unknown odor. Therefore, bees’ inability to use 1 s stimulus onset asynchrony for odor segregation probably does not reflect a limit in the temporal resolution of odor encoding. Rather, it might reflect a limit in temporal resolution of encoding the reward or of the molecular processes underlying odor-reward learning (Hammer, 1993; Menzel et al., 1996; Liu et al., 2012; Huetteroth et al., 2015).

In contrast, the finding that insects can use stimulus onset asynchronies of a few milliseconds for odor segregation when the odorant is known (Baker et al., 1998; Szyszka et al., 2012; Sehdev et al., 2019) indicates that the temporal resolution which odorants can be encoded with is higher for known than for unknown odorants. The temporal resolution for encoding a known odorant could be higher because a known odorant activates temporally precise pattern-recognition neurons that are tuned to the specific neuronal activity pattern evoked by the known odorant. Those pattern-recognition neurons could be lateral horn neurons in case of odorants with innate valence (Jefferis et al., 2007; Roussel et al., 2014; Strutz et al., 2014; Jeanne et al., 2018), and mushroom body output neurons in case of odorants with learned valence (Strube-Bloss et al., 2011; Aso et al., 2014; Hige et al., 2015), or in case of previously encountered odorants (Cassenaer and Laurent, 2007). Whether different odorants originate from the same or different sources could be detected by coincidence-detecting neurons that receive input from those pattern recognition neurons (Sehdev et al., 2019).

Our finding that stimulus onset asynchronies of 5 s or longer, but not of 1 s or shorter, improve odor segregation, is consistent with the hypothesis that for unknown odorants, odor segregation depends on sensory adaptation. Sensory adaptation was previously proposed to underlie odor-background segregation in vertebrates (Kadohisa and Wilson, 2006; Linster et al., 2007). Neurons in the piriform cortex (analogous to the mushroom bodies) adapt to a constant odorant stimulus but remain responsive to a novel odorant pulse that arrives a few tens of seconds after the onset of the background odorant (Wilson, 1998).

Similarly, insect olfactory receptor neurons and projection neurons (in *Drosophila*) adapt to a constant background odorant stimulus (de Bruyne et al., 2001; Bhandawat et al., 2007; Nagel and Wilson, 2011; Martelli et al., 2013; Cafaro, 2016) but they remain responsive to a novel odorant pulse that arrives a few seconds after the onset of the background odorant (Cafaro, 2016). In honey bees, neural responses start adapting within a few 100 ms [projection neurons: (Sachse and Galizia, 2002; Krofczik et al., 2009); Kenyon cells: (Szyszka et al., 2005; Farkhooi et al., 2013; Froese et al., 2014)]. Therefore, in honey bees, like in vertebrates, sensory adaptation could filter stable background odors while the neural responses to a novel odorant with an asynchronous onset would not be diminished, thus facilitating the segregation of a novel odorant with a later onset than the background.

### Limitations of the Study and Outlook

Because it is impossible to directly assess bees’ odorant perception, we measured bees’ capability to associate an odorant A with a sucrose reward during classical conditioning. We measured bees’ associative learning of A, as the “correct response” during a memory test (a bee responds to A, but not to a novel control odorant). Bees showed fewer correct responses when A was mixed with B during conditioning, and introducing an onset asynchrony of 5 or 20 s between the arrival of B and A increased the number of correct responses. We interpreted this increase in correct responses as an increase in bees’ capability to perceptually segregate A from the mixture. Note however, that bees also likely associated odor B with the sucrose reward. This B-sucrose association could have affected bees’ correct responses to A through generalization. The interval between the onset of odor B and of the sucrose reward varied between the different asynchronous mixtures, and might have affected bees’ associative learning of B [as is the case in trace conditioning, where there is a stimulus-free gap between the onsets of the odorant and the sucrose reward (Szyszka et al., 2011)]. Thus, learning of B might have differed across the different mixtures, and these differences in learning B might have also affected bees’ correct responses to A. The contribution of such differences in learning and differences in perception could be disentangled to some extent by analyzing how neural responses to the synchronous and asynchronous mixtures relate to the responses of A alone (Guerrieri et al., 2005).

To get a better understanding of the perceptual mechanisms of odor segregation, it will be important to determine how the chemical and physical properties of odor stimuli affect odor segregation: for example, whether odor similarity, concentration and mixture composition affect bees’ capability for odor segregation, and whether bees can also use stimulus offset asynchrony and temporal decorrelation for segregating odors—stimulus cues that humans use for segregating concurrent sounds (Carlyon, 2004).

## Data Availability

All datasets generated for this study are included in the manuscript and/or the Supplementary Files.

## Author Contributions

AS and PS conceptualized and designed the study and wrote the manuscript. AS performed the data collection and performed the statistical analysis. PS supervised the study.

## Conflict of Interest Statement

The authors declare that the research was conducted in the absence of any commercial or financial relationships that could be construed as a potential conflict of interest.

## References

[B1] Andersson M. N. Binyameen M. Sadek M. M. Schlyter F. (2011). Attraction modulated by spacing of pheromone components and anti-attractants in a bark beetle and a moth. J. Chem. Ecol. 37, 899–911. 10.1007/s10886-011-9995-3 21750948

[B2] Aso Y. Sitaraman D. Ichinose T. Kaun K. R. Vogt K. Belliart-Guérin G.. (2014). Mushroom body output neurons encode valence and guide memory-based action selection in *Drosophila*. Elife 3:e04580. 10.7554/eLife.04580 25535794 PMC4273436

[B3] Baker T. C. Fadamiro H. Y. Cosse A. A. (1998). Moth uses fine tuning for odour resolution. Nature 393, 530–530. 10.1038/31131

[B4] Bhandawat V. Olsen S. R. Gouwens N. W. Schlief M. L. Wilson R. I. (2007). Sensory processing in the Drosophila antennal lobe increases reliability and separability of ensemble odor representations. Nat. Neurosci. 10, 1474–1482. 10.1038/nn1976 17922008 PMC2838615

[B5] Bitterman M. E. Menzel R. Fietz A. Schäfer S. Schafer S. (1983). Classical conditioning of proboscis extension in honeybees (*Apis mellifera*). J. Comp. Psychol. 97, 107–119. 10.1037/0735-7036.97.2.107 6872507

[B6] Brill M. F. Rosenbaum T. Reus I. Kleineidam C. J. Nawrot M. P. Rossler W. (2013). Parallel processing via a dual olfactory pathway in the honeybee. J. Neurosci. 33, 2443–2456. 10.1523/JNEUROSCI.4268-12.2013 23392673 PMC6619158

[B7] Cafaro J. (2016). Multiple sites of adaptation lead to contrast encoding in the Drosophila olfactory system. Physiol. Rep. 4:e12762. 10.14814/phy2.12762 27053295 PMC4831330

[B8] Carlyon R. P. (2004). How the brain separates sounds. Trends Cogn. Sci. 8, 465–471. 10.1016/j.tics.2004.08.008 15450511

[B9] Cassenaer S. Laurent G. (2007). Hebbian STDP in mushroom bodies facilitates the synchronous flow of olfactory information in locusts. Nature 448, 709–713. 10.1038/nature05973 17581587

[B10] Celani A. Villermaux E. Vergassola M. (2014). Odor landscapes in turbulent environments. Phys. Rev. X 4:041015 10.1103/physrevx.4.041015

[B11] Chandra S. Smith B. H. (1998). An analysis of synthetic processing of odor mixtures in the honeybee (*Apis mellifera*). J. Exp. Biol. 201, 3113–3121. 9787131 10.1242/jeb.201.22.3113

[B12] de Bruyne M. Foster K. Carlson J. R. (2001). Odor coding in the antenna. Neuron 30, 537–552. 10.1016/S0896-6273(01)00289-6 11395013

[B13] Deisig N. Lachnit H. Giurfa M. Hellstern F. (2001). Configural olfactory learning in honeybees: negative and positive patterning discrimination. Learn. Mem. 8, 70–78. 10.1101/lm.8.2.70 11274252 PMC311365

[B14] Deisig N. Lachnit H. Sandoz J.-C. Lober K. Giurfa M. (2003). A modified version of the unique cue theory accounts for olfactory compound processing in honeybees. Learn. Mem. 10, 199–208. 10.1101/lm.55803 12773584 PMC202310

[B15] Egea-Weiss A. Renner A. Kleineidam C. J. Szyszka P. (2018). High precision of spike timing across olfactory receptor neurons allows rapid odor coding in drosophila. iScience 4, 76–83. 10.1016/j.isci.2018.05.009 30240755 PMC6147046

[B16] Erskine A. Ackels T. Dasgupta D. Fukunaga I. Schaefer A. T. (2019). Mammalian olfaction is a high temporal bandwidth sense. bioRxiv [Preprint] 570689.

[B17] Farkhooi F. Froese A. Muller E. Menzel R. Nawrot M. P. (2013). Cellular adaptation facilitates sparse and reliable coding in sensory pathways. PLoS Comput. Biol. 9:e1003251. 10.1371/journal.pcbi.1003251 24098101 PMC3789775

[B18] Froese A. Szyszka P. Menzel R. (2014). Effect of GABAergic inhibition on odorant concentration coding in mushroom body intrinsic neurons of the honeybee. J. Comp. Physiol. A Neuroethol. Sens. Neural Behav. Physiol. 200, 183–195. 10.1007/s00359-013-0877-8 24362942

[B19] Gerber B. Ullrich J. (1999). No evidence for olfactory blocking in honeybee classical conditioning. J. Exp. Biol. 202, 1839–1854. 10359686 10.1242/jeb.202.13.1839

[B20] Guerrieri F. Schubert M. Sandoz J. C. Giurfa M. (2005). Perceptual and neural olfactory similarity in honeybees. PLoS Biol. 3:e60. 10.1371/journal.pbio.0030060 15736975 PMC1043859

[B21] Hammer M. (1993). An identified neuron mediates the unconditioned stimulus in associative olfactory learning in honeybees. Nature 366, 59–63. 10.1038/366059a0 24308080

[B22] Hendin O. Horn D. Hopfield J. J. (1994). Decomposition of a mixture of signals in a model of the olfactory bulb. Proc. Natl. Acad. Sci. U S A 91, 5942–5946. 10.1073/pnas.91.13.5942 8016093 PMC44113

[B23] Hige T. Aso Y. Modi M. N. Rubin G. M. Turner G. C. (2015). Heterosynaptic plasticity underlies aversive olfactory learning in Drosophila. Neuron 88, 985–998. 10.1016/j.neuron.2015.11.003 26637800 PMC4674068

[B24] Hopfield J. F. Gelperin A. (1989). Differential conditioning to a compound stimulus and its components in the terrestrial mollusc Limax maximus. Behav. Neurosci. 103, 329–333. 10.1037/0735-7044.103.2.329

[B25] Hopfield J. J. (1991). Olfactory computation and object perception. Proc. Natl. Acad. Sci. U S A 88, 6462–6466. 10.1073/pnas.88.15.6462 1862075 PMC52105

[B26] Huetteroth W. Perisse E. Lin S. Klappenbach M. Burke C. Waddell S. (2015). Sweet taste and nutrient value subdivide rewarding dopaminergic neurons in drosophila. Curr. Biol. 25, 751–758. 10.1016/j.cub.2015.01.036 25728694 PMC4372253

[B28] Jeanne J. M. Fişek M. Wilson R. I. (2018). The organization of projections from olfactory glomeruli onto higher-order neurons. Neuron 98, 1198.e6–1213.e6. 10.1016/j.neuron.2018.05.011 29909998 PMC6051339

[B27] Jeanne J. M. Wilson R. I. (2015). Convergence, divergence, and reconvergence in a feedforward network improves neural speed and accuracy. Neuron 88, 1014–1026. 10.1016/j.neuron.2015.10.018 26586183 PMC5488793

[B29] Jefferis G. S. X. E. Potter C. J. Chan A. M. Marin E. C. Rohlfing T. Maurer C. R.. (2007). Comprehensive maps of drosophila higher olfactory centers: spatially segregated fruit and pheromone representation. Cell 128, 1187–1203. 10.1016/j.cell.2007.01.040 17382886 PMC1885945

[B30] Jinks A. Laing D. G. (1999). A limit in the processing of components in odour mixtures. Perception 28, 395–404. 10.1068/p2898 10615476

[B31] Kadohisa M. Wilson D. A. (2006). Olfactory cortical adaptation facilitates detection of odors against background. J. Neurophysiol. 95, 1888–1896. 10.1152/jn.00812.2005 16251260 PMC2292127

[B32] Korner-Nievergelt F. Roth T. von Felten S. Guelat J. Almasi B. Korner-Nievergelt P. (2015). Bayesian Data Analysis in Ecology Using Linear Models with R, BUGS, and Stan. London: Academic Press.

[B33] Kree M. Duplat J. Villermaux E. (2013). The mixing of distant sources. Phys. Fluids 25:091103 10.1063/1.4820015

[B34] Krofczik S. Menzel R. Nawrot M. P. (2009). Rapid odor processing in the honeybee antennal lobe network. Front. Comput. Neurosci. 2:9. 10.3389/neuro.10.009.2008 19221584 PMC2636688

[B35] Laloi D. Roger B. Blight M. M. Wadhams L. J. Pham-Delegue M. H. (1999). Individual learning ability and complex odor recognition in the honey bee, *Apis mellifera* L. J. Insect Behav. 12, 585–597. 10.1023/A:1020919501871

[B36] Laska M. Hudson R. (1993). Discriminating parts from the whole: determinants of odor mixture perception in squirrel monkeys, Saimiri sciureus. J. Comp. Physiol. A 173, 249–256. 10.1007/bf00192984 8410741

[B37] Linster C. Henry L. Kadohisa M. Wilson D. A. (2007). Synaptic adaptation and odor-background segmentation. Neurobiol. Learn. Mem. 87, 352–360. 10.1016/j.nlm.2006.09.011 17141533

[B38] Liu C. Plaçais P.-Y. Yamagata N. Pfeiffer B. D. Aso Y. Friedrich A. B.. (2012). A subset of dopamine neurons signals reward for odour memory in Drosophila. Nature 488, 512–516. 10.1038/nature11304 22810589

[B39] Lynn W. H. Meyer E. A. Peppiatt C. E. Derby C. D. (1994). Perception of odor mixtures by the spiny lobster Panulirus argus. Chem. Senses 19, 331–347. 10.1093/chemse/19.4.331 7812726

[B40] Martelli C. Carlson J. Emonet T. (2013). Intensity invariant dynamics and odor-specific latencies in olfactory receptor neuron response. J. Neurosci. 33, 6285–6297. 10.1523/JNEUROSCI.0426-12.2013 23575828 PMC3678969

[B41] Menzel R. Hammer M. Müller U. Rosenboom H. (1996). Behavioral, neural and cellular components underlying olfactory learning in the honeybee. J. Physiol. Paris 90, 395–398. 10.1016/s0928-4257(97)87928-4 9089522

[B42] Müller D. Abel R. Brandt R. Zöckler M. Menzel R. (2002). Differential parallel processing of olfactory information in the honeybee, *Apis mellifera* L. J. Comp. Physiol. A Neuroethol. Sens. Neural Behav. Physiol. 188, 359–370. 10.1007/s00359-002-0310-1 12073081

[B43] Müller D. Gerber B. Hellstern F. Hammer M. Menzel R. (2000). Sensory preconditioning in honeybees. J. Exp. Biol. 203, 1351–1364. 10729283 10.1242/jeb.203.8.1351

[B44] Murlis J. Elkinton J. Carde R. (1992). Odor plumes and how insects use them. Annu. Rev. Entomol. 37, 505–532. 10.1146/annurev.ento.37.1.505

[B46] Nagel K. I. Wilson R. I. (2011). Biophysical mechanisms underlying olfactory receptor neuron dynamics. Nat. Neurosci. 14, 208–216. 10.1038/nn.2725 21217763 PMC3030680

[B47] Nikonov A. A. Leal W. S. (2002). Peripheral coding of sex pheromone and a behavioral antagonist in the Japanese beetle, Popillia japonica. J. Chem. Ecol. 28, 1075–1089. 10.1023/A:1015274104626 12049228

[B48] Paoli M. Albi A. Zanon M. Zanini D. Antolini R. Haase A. (2018). Neuronal response latencies encode first odor identity information across subjects. J. Neurosci. 38, 9240–9251. 10.1523/JNEUROSCI.0453-18.2018 30201774 PMC6705991

[B49] Pavlov I. P. (1927). Conditioned Reflexes: An Investigation of the Physiological Activity of the Cerebral Cortex., ed. G. Valsievich Anrep. London: Oxford University Press. 10.5214/ans.0972-7531.1017309 PMC4116985 25205891

[B50] Pelz C. Gerber B. Menzel R. (1997). Odorant intensity as a determinant for olfactory conditioning in honeybees: roles in discrimination, overshadowing and memory consolidation. J. Exp. Biol. 200, 837–847. 9076967 10.1242/jeb.200.4.837

[B51] Quinn W. G. Harris W. A. Benzer S. (1974). Conditioned behavior in *Drosophila melanogaster*. Proc. Natl. Acad. Sci. U S A 71, 708–712. 10.1073/pnas.71.3.708 4207071 PMC388082

[B52] Raiser G. Galizia C. G. G. Szyszka P. (2016). A high-bandwidth dual-channel olfactory stimulator for studying temporal sensitivity of olfactory processing. Chem. Senses 42, 141–151. 10.1093/chemse/bjw114 27988494

[B100] R Core Team (2017). R: A Language and Environment for Statistical Computing. Vienna, Austria: R Foundation for Statistical Computing. Available online at: http://www.r-project.org

[B53] Reinhard J. Sinclair M. Srinivasan M. V. Claudianos C. (2010). Honeybees learn odour mixtures *via* a selection of key odorants. PLoS One 5:e9110. 10.1371/journal.pone.0009110 20161714 PMC2817008

[B54] Riffell J. A. Shlizerman E. Sanders E. Abrell L. Medina B. Hinterwirth A. J.. (2014). Sensory biology. Flower discrimination by pollinators in a dynamic chemical environment. Science 344, 1515–1518. 10.1126/science.1251041 24970087

[B55] Rokni D. Hemmelder V. Kapoor V. Murthy V. N. (2014). An olfactory cocktail party: figure-ground segregation of odorants in rodents. Nat. Neurosci. 17, 1225–1232. 10.1038/nn.3775 25086608 PMC4146660

[B56] Roussel E. Carcaud J. Combe M. Giurfa M. Sandoz J.-C. (2014). Olfactory coding in the honeybee lateral horn. Curr. Biol. 24, 561–567. 10.1016/j.cub.2014.01.063 24560579

[B57] Sachse S. Galizia C. G. (2002). Role of inhibition for temporal and spatial odor representation in olfactory output neurons: a calcium imaging study. J. Neurophysiol. 87, 1106–1117. 10.1152/jn.00325.2001 11826074

[B58] Sachse S. Rappert A. Galizia C. G. (1999). The spatial representation of chemical structures in the antennal lobe of honeybees: steps towards the olfactory code. Eur. J. Neurosci. 11, 3970–3982. 10.1046/j.1460-9568.1999.00826.x 10583486

[B59] Saha D. Leong K. Li C. Peterson S. Siegel G. Raman B. (2013). A spatiotemporal coding mechanism for background-invariant odor recognition. Nat. Neurosci. 16, 1830–1839. 10.1038/nn.3570 24185426

[B60] Sehdev A. Mohammed Y. G. Triphan T. Szyszka P. (2019). Olfactory object recognition based on fine-scale stimulus timing in *Drosophila*. iScience 13, 113–124. 10.1016/j.isci.2019.02.014 30826726 PMC6402261

[B61] Smith B. (1998). Analysis of interaction in binary odorant mixtures. Physiol. Behav. 65, 397–407. 10.1016/s0031-9384(98)00142-5 9877404

[B62] Smith B. H. Cobey S. (1994). The olfactory memory of the honeybee *Apis mellifera*. II. Blocking between odorants in binary mixtures. J. Exp. Biol. 195, 91–108. 7964421 10.1242/jeb.195.1.91

[B63] Soltys M. A. A. Crimaldi J. P. P. (2015). Joint probabilities and mixing of isolated scalars emitted from parallel jets. J. Fluid Mech. 769, 130–153. 10.1017/jfm.2015.113

[B64] Staubli U. Fraser D. Faraday R. Lynch G. (1987). Olfaction and the “data” memory system in rats. Behav. Neurosci. 101, 757–765. 10.1037//0735-7044.101.6.757 3426792

[B65] Stevenson R. J. Wilson D. A. (2007). Odour perception: an object-recognition approach. Perception 36, 1821–1833. 10.1068/p5563 18283932

[B66] Strube-Bloss M. F. Nawrot M. P. Menzel R. (2011). Mushroom body output neurons encode odor-reward associations. J. Neurosci. 31, 3129–3140. 10.1523/jneurosci.2583-10.2011 21414933 PMC6623757

[B67] Strutz A. Soelter J. Baschwitz A. Farhan A. Grabe V. Rybak J.. (2014). Decoding odor quality and intensity in the *Drosophila* brain. Elife 3:e04147. 10.7554/elife.04147 25512254 PMC4270039

[B68] Szyszka P. Demmler C. Oemisch M. Sommer L. Biergans S. Birnbach B.. (2011). Mind the gap: olfactory trace conditioning in honeybees. J. Neurosci. 31, 7229–7239. 10.1523/jneurosci.6668-10.2011 21593307 PMC6622586

[B69] Szyszka P. Ditzen M. Galkin A. Galizia C. G. G. Menzel R. (2005). Sparsening and temporal sharpening of olfactory representations in the honeybee mushroom bodies. J. Neurophysiol. 94, 3303–3313. 10.1152/jn.00397.2005 16014792

[B70] Szyszka P. Stierle J. S. Biergans S. Galizia C. G. (2012). The speed of smell: odor-object segregation within milliseconds. PLoS One 7:e36096. 10.1371/journal.pone.0036096 22558344 PMC3338635

[B71] Weissburg M. Atkins L. Berkenkamp K. Mankin D. (2012). Dine or dash? Turbulence inhibits blue crab navigation in attractive-aversive odor plumes by altering signal structure encoded by the olfactory pathway. J. Exp. Biol. 215, 4175–4182. 10.1242/jeb.077255 23136153

[B72] Wilson D. A. (1998). Synaptic correlates of odor habituation in the rat anterior piriform cortex. J. Neurophysiol. 80, 998–1001. 10.1152/jn.1998.80.2.998 9705487

[B73] Wright G. A. Smith B. H. (2004). Variation in complex olfactory stimuli and its influence on odour recognition. Proc. Biol. Sci. 271, 147–152. 10.1098/rspb.2003.2590 15058390 PMC1691576

